# Impact of Encapsulated Iron Availability on the Growth Kinetics of *Campylobacter jejuni*

**DOI:** 10.3390/pathogens15040400

**Published:** 2026-04-07

**Authors:** Elena G. Olson, Emily A. Matiak, Joshua A. Jendza, Steven C. Ricke

**Affiliations:** 1 Meat Science & Animal Biologics Discovery Program, Department of Animal and Dairy Sciences, University of Wisconsin-Madison, 1933 Observatory Drive, Madison, WI 53706, USA; egolson2@wisc.edu (E.G.O.); matiak@wisc.edu (E.A.M.); 2 Qualitech LLC, 318 Lake Hazeltine Drive, Chaska, MN 55318, USA; joshuaj@qualitechco.com

**Keywords:** *Campylobacter jejuni*, iron bioavailability, encapsulated iron, SQM^®^ Iron, poultry microbiota, pathogen proliferation, controlled-release minerals, iron-limited conditions, gut health, foodborne pathogens

## Abstract

Background: *Campylobacter jejuni*, a leading foodborne pathogen in poultry, relies heavily on iron for survival and colonizes the gastrointestinal tract (GIT). Iron supplementation in poultry diets can inadvertently promote pathogen growth, particularly when excess or poorly absorbed iron accumulates in the lower GIT. Encapsulated iron products, such as SQM^®^ Iron, offer a controlled-release mechanism that may mitigate this risk by reducing iron availability to microbes. Objective: This study evaluated the effects of free (FeSO_4_) versus polysaccharide–iron complex (PIC) on *C. jejuni* growth under iron-limited conditions, hypothesizing that encapsulated iron would support slower and more limited bacterial proliferation due to delayed iron release. Methods: Growth kinetics of *C. jejuni* ATCC 700819 were assessed in chelated Mueller–Hinton broth supplemented with three iron concentrations (10, 20, and 50 ppm) of FeSO_4_, PIC, or PIC matrix without iron. Optical density was measured every 20 min over 48 h under microaerophilic conditions. Maximum growth rate (µmax) and carrying capacity (K) were derived using non-linear curve modeling. ANOVA evaluated statistical significance with Tukey’s HSD post hoc comparisons. Results: Free iron (FeSO_4_) consistently supported the highest µmax and K values across both trials, indicating rapid and robust *C. jejuni* proliferation. The effect of encapsulated iron was variable: at higher concentrations (50 ppm) it approached FeSO_4_ performance, but at lower concentrations (10 ppm) its effect differed markedly between trials, sometimes supporting growth comparable to free iron and sometimes supporting substantially slower growth. The PIC matrix alone did not promote growth. These variable results indicate that the relationship between encapsulated iron and *C. jejuni* proliferation is complex and concentration-dependent. Conclusions: Free iron consistently promotes robust *C. jejuni* growth due to immediate bioavailability. The impact of encapsulated iron on *C. jejuni* proliferation is nuanced and variable, particularly at lower concentrations, suggesting its role in pathogen control is not straightforward and requires further investigation under controlled conditions. Furthermore, in vivo research is warranted to validate its utility in poultry pathogen management strategies.

## 1. Introduction

Iron is an essential nutrient for *Campylobacter* growth, playing a crucial role in its metabolism, survival, and colonization within the host gastrointestinal tract (GIT) [1,2]. The ability of *Campylobacter* to acquire and utilize iron is vital for its proliferation, as it lacks the robust intrinsic mechanisms for survival in iron-limited environments. Among foodborne pathogens, *Campylobacter*, particularly *C. jejuni* and *C. coli* is a leading cause of bacterial gastroenteritis worldwide, with poultry serving as a primary reservoir [3,4,5]. The bacterium relies on environmental iron sources, including siderophores such as enterobactin, which the GIT microbiota produces to facilitate iron uptake [6]. Genome sequencing has revealed that *C. jejuni* possesses multiple iron acquisition systems, enabling it to adapt to fluctuating iron availability in the gastrointestinal tract (GIT) [7,8]. Iron is a critical dietary component for animals, necessary for key physiological functions such as oxygen transport, enzyme activity, and immune system support [8,9]. Iron deficiency can lead to anemia, weakened immune response, and reduced growth performance, particularly in livestock and poultry. However, excessive free iron can also pose risks, including fostering the overgrowth of pathogenic bacteria in the gut [9,10]. Therefore, a controlled iron supplementation strategy is crucial for optimizing animal health while minimizing the risk of pathogen proliferation.

Iron is highly bioavailable in its free form, and its regulation in microbial growth systems presents challenges, as excess free iron can promote pathogen overgrowth. In animals, this dysbiosis may trigger inflammatory responses in the gastrointestinal tract (GIT), resulting in diarrhea, nausea, and vomiting [9]. Additionally, the overgrowth of pathogenic bacteria can impair gut barrier function, compromise immune defenses, and ultimately lead to poor overall health and diminished growth performance [9,10]. Given *Campylobacter*’s reliance on iron, a targeted approach for limiting its access to this resource could reduce its persistence in poultry and mitigate foodborne transmission.

Encapsulated iron, such as the SQM^®^ Iron (QualiTech, Chaska, MN, USA) used in this study, offers a controlled-release mechanism that modulates iron availability and microbial growth dynamics. A polysaccharide–iron complex (PIC) is a compound formed by iron ions and polysaccharide as a carrier [11]. This mechanism ensures that iron remains intact through the digestive system, shielding it from antagonistic interactions and degradation. The encapsulated iron is then precisely delivered at the site of absorption in the small intestine, improving its bioavailability to the host animal [11]. Additionally, this controlled release reduces iron availability to pathogenic bacteria, such as *Campylobacter*, *Salmonella*, and *E. coli*, thereby supporting better gut health and overall animal performance [11]. Unlike free iron, which is immediately accessible for bacterial growth, PIC provides a gradual and regulated release, making it a promising alternative for balancing nutritional needs and microbial control. Given *Campylobacter*’s dependence on iron for survival, understanding the effects of encapsulated iron on its growth is essential for developing targeted interventions.

The current study aimed to assess the impact of PIC iron on *Campylobacter* growth compared to free iron and iron-limited conditions. The study hypothesized that encapsulated iron would result in slower *Campylobacter* growth than free iron due to its controlled-release properties. However, at higher concentrations, encapsulated iron would approximate the growth-supporting capabilities of free iron, though with a moderated carrying capacity. While encapsulated iron can slowly release iron, higher concentrations of released iron can support *Campylobacter* growth, similarly to free iron. However, because the release is gradual, the carrying capacity should decline compared to the free iron. The current study sought to provide insight into potential strategies for limiting *Campylobacter* colonization in poultry and reducing its risk as a foodborne pathogen by investigating these dynamics.

## 2. Methods

### 2.1. Bacterial Strain and Culture Conditions

*Campylobacter* subsp. *jejuni* ATCC 700819 was used in the current study. *C. jejuni* was stored at −80 °C in Tryptic Soy Broth (Sigma-Aldrich, Rochester, NY, USA) containing 20% (*v*/*v*) glycerol. A frozen stock of *C. jejuni* was streak-plated from the −80 °C vials on modified charcoal–cefoperazone–deoxycholate agar (mCCDA; Millipore, Himedia, Mumbai, India) and incubated under microaerophilic conditions (5% O_2_, 10% CO_2_, and 85% N_2_) at 42 °C for 24 h. Next, a single colony of *C. jejuni* was inoculated into 40 mL Bolton broth (Sigma-Aldrich, Neogen, Lansing, MI, USA) with lysed horse blood following the manufacturer’s instructions. The cultures were incubated under microaerophilic conditions at 42 °C with constant shaking at 100 revolutions per minute (RPM) for 48 h. The culture was then pelleted and washed five times with PBS to eliminate the traces of blood. Subsequently, the pellet was resuspended in Mueller-Hinton broth (MHB) and incubated at 42 °C for 48 h before the onset of the study.

### 2.2. Chelation of Iron in Mueller Hinton for the Study

A 2× concentration of MHB was prepared by dissolving double the standard amount of dehydrated medium in 1 L of deionized (DI) water, followed by autoclaving. The sterilized broth was stored at 0 °C until it was used. To chelate available iron and create iron-limited conditions, a 2,2-dipyridyl (DIP) stock solution was prepared by dissolving 42.6 mg of solid DIP in 100 mL of DI water with continuous stirring until fully dissolved, yielding a final concentration of 0.426 mg/mL. This solution was added to 2× MHB to achieve a final working concentration of 21.3 µg/mL, equivalent to approximately 0.11 mM DIP (based on a molecular weight of 156.19 g/mol). This DIP concentration was selected based on preliminary experiments, demonstrating significant inhibition of *Campylobacter jejuni* growth. Specifically, this concentration reduced the maximum specific growth rate (μmax) and carrying capacity while also extending the lag phase, indicating effective iron chelation and growth suppression.

### 2.3. Iron Treatments

To investigate the impact of iron on the growth kinetics of *Campylobacter jejuni*, three treatments were utilized: (1) a positive control that consisted of the free iron (FeSO_4_·H_2_O), (2) encapsulated iron with PolyTransport technology (PIC, QualiTech), and (3) a negative control that included the PIC matrix without iron. The independent variables in this experiment were the type of iron treatment (free iron, PIC-Fe, and PIC without Fe) and the concentration of iron (10, 20, and 50 ppm). Treatments were combined with DIP-chelated 2× MHB and dispensed into 96-well plates. The experiment was conducted in two independent biological trials (Trial 1 and Trial 2), each representing a replicate performed on separate days with freshly prepared inocula. Within each trial, each treatment to concentration combination was represented by three technical replicates (individual wells) arranged on the 96-well plate. The initial inoculum concentration was verified prior to each trial by serial dilution and CFU plating on mCCDA agar. Each well was inoculated with approximately 10^2^ CFU of *C. jejuni* and incubated for 48 h at 42 °C under microaerophilic conditions.

**Figure 1:**
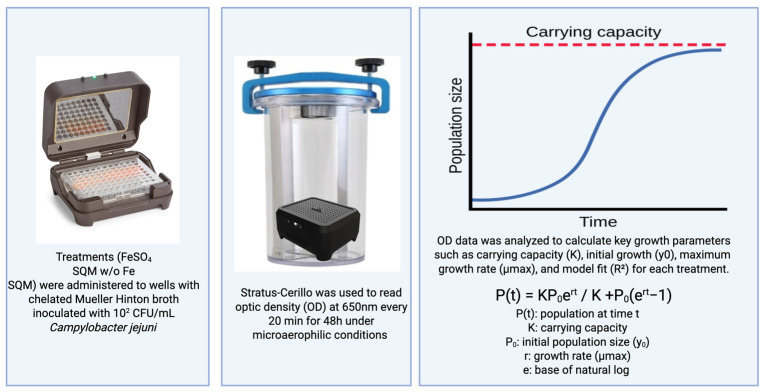
Overview of the experimental design in the current study. Created in BioRender. 2025. Available online: https://BioRender.com/dpgra9s (accessed on 2 March 2026).

The dependent variable was the growth kinetics of *C. jejuni*, which was monitored by measuring the optical density (OD) at 650 nm at 20-min intervals. OD data were analyzed in R Studio (version 2025.05.0+496, Posit PBC, Boston, MA, USA) using the growth_rates package to calculate key growth parameters, including carrying capacity (K), initial growth (y_0_), maximum growth rate (µmax), and model fit (R^2^), for each treatment. Figure 1 describes the overview of the experimental design in the current study.

## 3. Statistical Analysis

*Growth Rate and Carrying Capacity Estimation Using Logistic Growth Modeling*

Growth dynamics of *Campylobacter jejuni* were monitored in iron-supplemented Mueller Hinton broth using OD650 readings recorded every 20 min over 48 h under microaerophilic conditions. Readings were obtained using the Stratus-Cerillo system within a controlled anaerobic environment. Each treatment—FeSO_4_ (free iron), PIC-Fe (encapsulated), and PIC matrix without Fe—was inoculated at ~10^2^ CFU/mL and monitored for kinetic changes in population growth. Optical density data were analyzed in R Studio using the growthrates package, which applies a logistic model commonly used in microbial ecology and population dynamics [12]. The logistic growth function used was:

P(t) = KP_0_e^rt^/K + P_0_(e^rt^ − 1)

where P(t) is the population (OD_650_) at time t

K is the carrying capacity

P_0_ is the initial population size (y_0_)

r is the maximum growth rate (μmax)

e is the base of the natural logarithm

The model was fit to each replicate’s time series using non-linear least squares, with parameter estimation for: (1) carrying capacity (K) at the asymptotic maximum OD650, (2) the maximum specific growth rate, estimated at the curve’s inflection point (μmax, r) and (3) initial population size (y_0_ or P_0_), interpolated from early OD readings. Goodness of fit was evaluated using R^2^ values.

It should be noted that the logistic model may not be appropriate for all treatment conditions; specifically, the PIC-matrix-only treatment at 50 ppm, the fitted parameters (high µmax, low K) may reflect a non-sigmoidal optical density trajectory or transient optical artifacts rather than true logistic growth dynamics. Caution is warranted when interpreting fitted parameters in cases where model assumptions may not hold. OD readings were truncated prior to 48 h once cultures reached stationary phase, as inclusion of the full-time course reduced graph clarity without adding interpretive value. Fitted parameters were exported and used for treatment comparisons. Data wrangling and plotting were performed using tidyverse and ggplot2 packages (version 2025.05.0+496, Posit PBC, Boston, MA, USA).

## 4. Results

### 4.1. Logistic Growth Modeling Captured Treatment-Dependent Differences in C. jejuni Growth

To evaluate how iron source and concentration shaped *C. jejuni* proliferation under iron-limited conditions, OD650 growth curves from both trials were fit with a logistic growth model and compared using maximum growth rate (μmax), carrying capacity (K), and model fit (R^2^). The model fit was generally high across trials making kinetic parameters suitable for treatment comparison. The main effects were interpreted across iron source and concentration. Across treatments, the clearest biological differences were observed in μmax and K. The estimated growth kinetic parameters across treatments and iron concentrations are summarized in Table 1, Table 2 and Table 3.

### 4.2. Low Iron Availability Revealed the Greatest Divergence Between Free and Encapsulated Iron Responses

**Figure 2:**
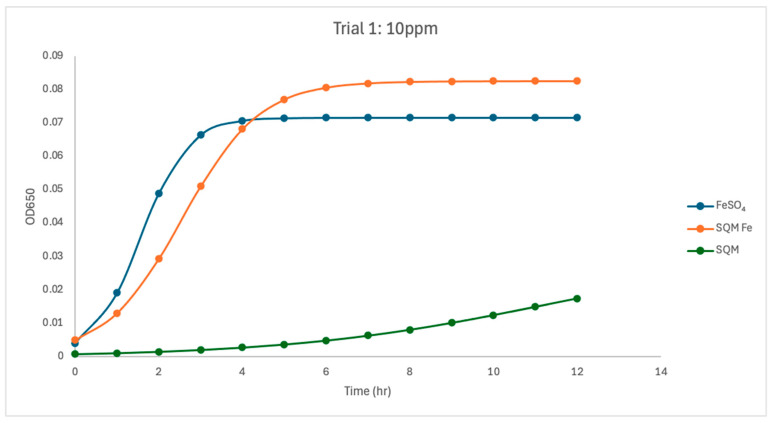
Growth dynamics for Trial 1 at 10 ppm. Free iron (Fe) treatment demonstrated the fastest growth rate (µmax = 1.78), indicating optimal iron availability for *Campylobacter* growth. Encapsulated iron (PIC) showed the highest carrying capacity (K = 0.0826) but slower growth (µmax = 0.80), reflecting delayed iron release. PIC without iron resulted in minimal growth (K = 0.032, µmax = 0.40), underscoring the critical role of iron in microbial growth. Growth was predictable in all treatments, as evidenced by high R^2^ values.

**Figure 3:**
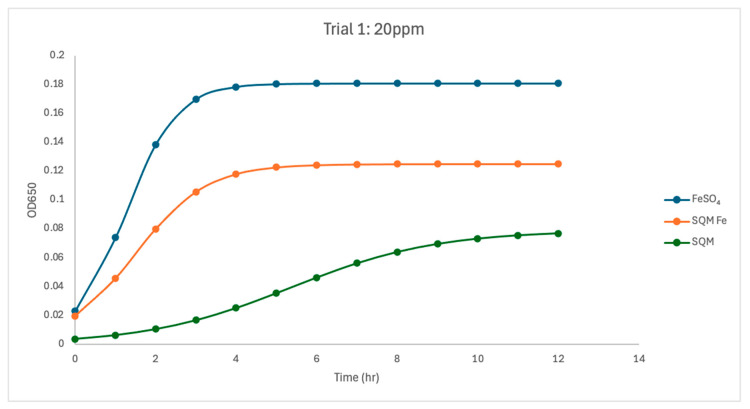
Growth dynamics for Trial 1 at 20 ppm. Free iron supported the highest carrying capacity (K = 0.1809) and a strong growth rate (µmax = 1.55). Encapsulated iron exhibited a moderate K (K = 0.1251) and slower growth (µmax = 1.13), with delayed iron release limiting bioavailability. PIC without iron showed limited growth (K = 0.08, µmax = 0.55), further confirming iron’s necessity. Growth patterns remained consistent, as indicated by high R^2^ values across treatments.

**Figure 4:**
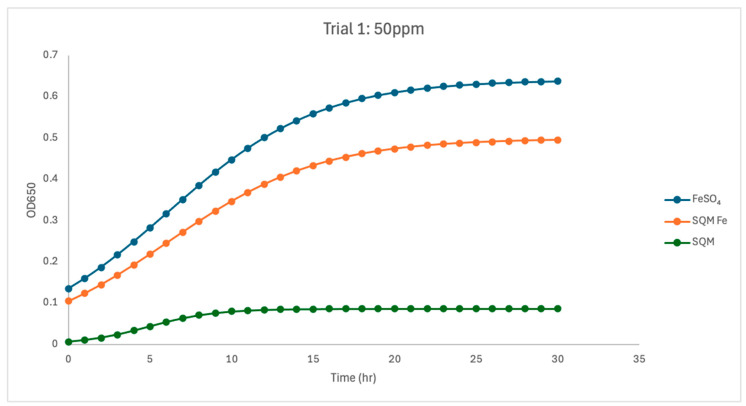
Growth dynamics for Trial 1 at 50 ppm. Free iron exhibited the highest carrying capacity (K = 0.6416) and an increased growth rate (µmax = 1.21), supporting robust microbial populations. Encapsulated iron closely approached free iron performance (K = 0.4985, µmax = 0.98), with delayed release still evident. PIC without iron sustained minimal growth (K = 0.116, µmax = 0.721), emphasizing iron’s indispensable role. High R^2^ values across treatments reflected predictable and consistent growth dynamics.

**Figure 5:**
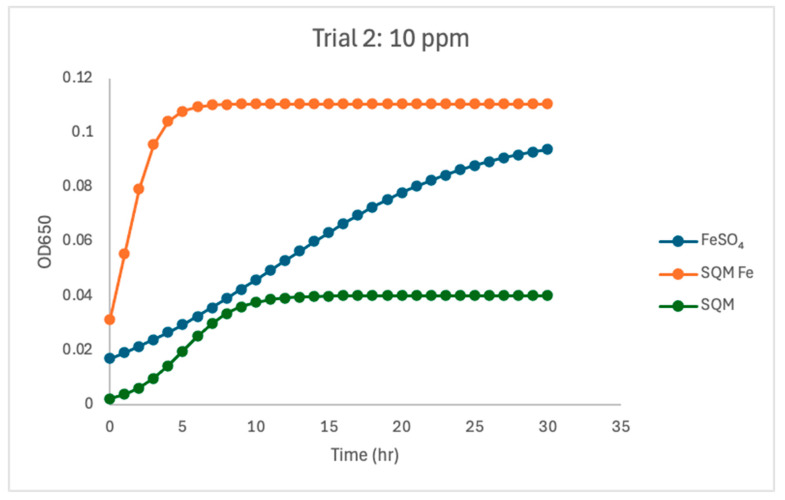
Growth dynamics for Trial 2 at 10 ppm. Encapsulated iron (PIC) treatment supported the fastest *C. jejuni* growth (µmax = 0.92) and the highest carrying capacity (K = 0.11). Free iron (FeSO_4_) exhibited a slower growth rate (µmax = 0.92) and a slightly lower carrying capacity (K = 0.10). PIC without iron resulted in a surprisingly high growth rate (µmax = 0.55) but a low carrying capacity (K = 0.040), underscoring the importance of iron availability Growth was predictable in all treatments, as evidenced by high R^2^ values.

At 10 ppm, the iron source had the strongest and most variable effect on *C. jejuni* growth kinetics across the study. In Trial 1, free iron (FeSO_4_) supported the fastest growth (µmax = 1.78), clearly exceeding both encapsulated PIC-Fe (µmax = 0.80) and the matrix-only treatment (µmax = 0.40). Encapsulated PIC-Fe, however, achieved a carrying capacity comparable to or slightly greater than FeSO_4_ (K = 0.0826 versus 0.072), indicating that although *Campylobacter* reached similar final biomass, growth initiation was slower under encapsulated iron. In contrast, the matrix-only treatment remained growth-limited, with the lowest carrying capacity (K = 0.032) and substantially lower overall growth than either iron-containing treatment (Figure 2, Figure 3 and Figure 4). In Trial 2, the 10 ppm response differed notably from that in Trial 1 (Figure 5). Under these conditions, FeSO_4_ and PIC-Fe showed more similar behavior (µmax = 0.92 for both) and carrying capacities (K = 0.101 for FeSO_4_ and K = 0.101 for PIC-Fe). The matrix-only treatment again supported reduced growth, with a lower fitted growth rate (µmax = 0.55) and carrying capacity (K = 0.040) than either iron-containing treatment. This low-iron condition, therefore, represented the clearest threshold at which iron accessibility shaped growth behavior.

Within Trial 1, treatment type significantly affected maximum growth rate at 10 ppm (ANOVA, *p* < 0.05), indicating that iron source strongly influenced early *C. jejuni* proliferation under low-iron conditions. Pairwise comparisons showed that the PIC matrix without iron had a significantly lower µmax than free iron (*p* < 0.05). Carrying capacity was not significantly different among treatments at 10 ppm in Trial 1, suggesting that the strongest treatment effect at this concentration was on growth rate rather than on final biomass. In Trial 2, treatment type did not produce the same strong separation in µmax at 10 ppm. However, carrying capacity was significantly affected by treatment across all concentrations, including 10 ppm (ANOVA, *p* < 0.05). As in Trial 1, the matrix-only treatment exhibited lower fitted growth than the iron-containing treatments.

Between-trial analysis further confirmed that 10 ppm was the most variable concentration. For FeSO_4_, µmax differed significantly between Trial 1 and Trial 2 (ANOVA, *p* = 0.0035), with Trial 1 showing substantially faster growth than Trial 2 (Table 1). Carrying capacity for FeSO_4_ also trended higher in Trial 2 than in Trial 1 (*p* = 0.0538). R^2^ values for FeSO_4_ increased from Trial 1 to Trial 2, but this change was also not significant (*p* = 0.0833). In contrast, PIC-Fe did not differ significantly between trials for µmax, K, or R^2^ at 10 ppm (*p* > 0.17 for all comparisons), despite visible variability in fitted values. The matrix-only treatment also showed no significant between-trial differences in µmax, K, or R^2^ (*p* > 0.27), although absolute growth remained low in both trials. Together, the 10 ppm condition indicated that low iron supply was the most sensitive concentration for revealing differences in iron accessibility. Additionally, the lack of consistent reciprocity across trials suggests that small shifts in iron release or adaptation may strongly influence growth under restrictive conditions.

### 4.3. Intermediate Iron Availability Shifted Growth Responses Without Fully Resolving Differences Between Iron Sources

At 20 ppm, both iron-containing treatments supported substantial *C. jejuni* growth, although differences between free and encapsulated iron remained evident. In Trial 1, free iron supported the strongest overall growth dynamics (µmax = 1.55) and the highest carrying capacity (K = 0.181). Encapsulated PIC-Fe also supported growth at this concentration, with a lower growth rate (µmax = 1.13) and reduced carrying capacity (K = 0.125). The matrix-only treatment remained clearly lower, with reduced growth rate (µmax = 0.55) and carrying capacity (K = 0.079). A similar overall pattern was observed in Trial 2, although the difference between FeSO_4_ and PIC-Fe was smaller than in Trial 1. Free iron again supported strong growth, with a mean µmax of 2.23 and K of 0.162. Encapsulated PIC-Fe produced a similar response, with a nearly identical growth rate (µmax = 2.25) and a moderately lower carrying capacity (K = 0.130). In contrast, the matrix-only treatment again supported substantially reduced growth (µmax = 0.33, K = 0.048).

Treatment effects shifted from primarily influencing growth rate to also influencing biomass accumulation. In Trial 1, treatment type showed a trend toward affecting µmax at 20 ppm (ANOVA, *p* = 0.06), and carrying capacity was significantly affected by treatment (*p* < 0.05). Pairwise comparisons indicated that the PIC matrix without iron had a significantly lower K than free iron (*p* < 0.05). In Trial 2, treatment type significantly affected growth rate (ANOVA, *p* < 0.05). Pairwise comparisons showed that the matrix-only treatment had a significantly lower growth rate than the free-iron treatment (*p* < 0.05). Carrying capacity was also significantly affected by treatment in Trial 2 at all concentrations, including 20 ppm, again with the matrix-only treatment showing significantly lower K than free iron (*p* < 0.05).

Between-trial analysis showed that FeSO_4_ was relatively stable at 20 ppm, with no significant differences between Trial 1 and Trial 2 for µmax, K, or R^2^ (*p* > 0.12 for all comparisons). PIC-Fe also showed no significant differences in K or R^2^ (*p* > 0.84). Growth rate, however, increased from 1.13 in Trial 1 to 2.25 in Trial 2 and approached significance (*p* = 0.0517), suggesting greater variability in growth rate under encapsulated iron at this concentration. For the matrix-only treatment, µmax did not differ significantly between trials (*p* = 0.0566); K was significantly lower in Trial 2 than in Trial 1 (*p* = 0.0393), while R^2^ showed a near-significant decline (*p* = 0.0586). Overall, the 20 ppm condition represented an intermediate response state in which encapsulated iron supported substantial growth, but source-dependent differences in growth kinetics and biomass accumulation remained evident across and within trials.

### 4.4. High Iron Availability Supported Growth in Both Iron-Containing Treatments; However, Encapsulated Iron Exhibited Distinct Kinetic Properties Compared with Free Iron

At 50 ppm, both iron-containing treatments resulted in substantial *C. jejuni* growth, yet notable differences in growth parameters persisted between free and encapsulated iron. In Trial 1, free iron (FeSO_4_) yielded the highest overall growth (µmax = 0.216) and a carrying capacity (K = 0.642). Encapsulated PIC-Fe produced a comparable growth rate (µmax = 0.214) with a lower carrying capacity (K = 0.499), indicating reduced final biomass compared to free iron. The matrix-only treatment resulted in notably lower carrying capacity (K = 0.087) despite a higher fitted µmax (0.491), suggesting that transient optical changes occurred without sustained biomass production. In Trial 2, differences between FeSO_4_ and PIC-Fe persisted. Free iron supported a robust growth under high iron availability (µmax = 0.213, K = 0.803). Encapsulated PIC-Fe exhibited a lower growth rate (µmax = 0.126) and a similar carrying capacity (K = 0.852). The matrix-only treatment again resulted in lower overall growth (µmax = 0.345, K = 0.069). Within-trial statistical analyses corroborated these findings. In both trials, treatment type significantly influenced growth rate (ANOVA, *p* < 0.05), with pairwise comparisons indicating that the matrix-only treatment had a significantly lower µmax than both free iron and PIC-Fe (*p* < 0.05). Carrying capacity was also significantly affected by treatment in both trials (*p* < 0.05); the matrix-only and PIC-Fe treatments exhibited lower K values than free iron (*p* < 0.05). These findings demonstrate that increased iron concentration promoted growth in both FeSO_4_ and PIC-Fe treatments but did not eliminate the kinetic differences between free and encapsulated iron.

Between-trial analyses indicated that free iron was highly reproducible at 50 ppm. For FeSO_4_, no significant differences were observed between Trial 1 and Trial 2 in µmax, K, or R^2^ (*p* = 0.967, 0.967, and 0.446, respectively), reflecting consistent growth behavior under high free-iron conditions. In contrast, PIC-Fe demonstrated significant between-trial differences in both µmax and K. The mean µmax decreased from 0.195 in Trial 1 to 0.125 in Trial 2 (ANOVA, *p* = 0.0098), while K increased from 0.499 to 0.852 (*p* = 3.94 × 10^−6^). R^2^ did not differ significantly between trials (*p* = 0.118), indicating that model fit remained robust despite changes in fitted parameters. The matrix-only treatment showed no significant between-trial differences in µmax, K, or R^2^ at 50 ppm (*p* > 0.12 for all comparisons), although absolute growth remained low compared to the iron-containing treatments.

## 5. Discussion: Iron Bioavailability and *Campylobacter* Growth

### 5.1. Free Iron Enhances Campylobacter Growth Through Immediate Availability

**Figure 6:**
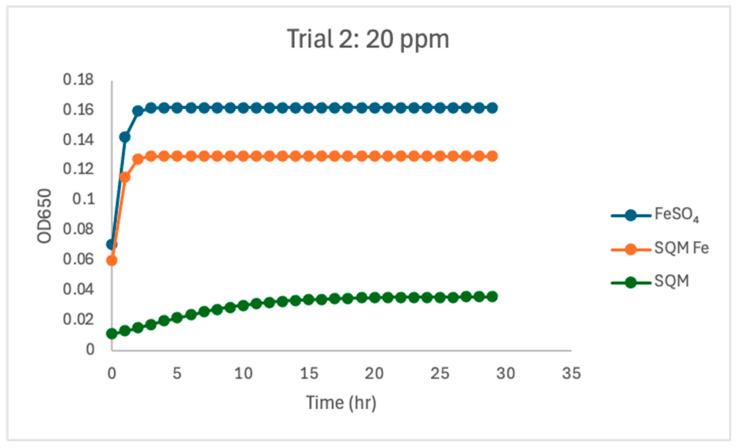
Growth dynamics for Trial 2 at 20 ppm. At 20 ppm, free iron supported the most vigorous growth dynamics (µmax = 2.23, K = 0.162). Encapsulated iron demonstrated a moderate carrying capacity (K = 0.129) and a high growth rate (µmax = 2.24), consistent with delayed iron release. PIC without iron showed limited growth (µmax = 0.245, K = 0.036). Growth patterns remained consistent, as indicated by high R^2^ values across treatments.

**Figure 7:**
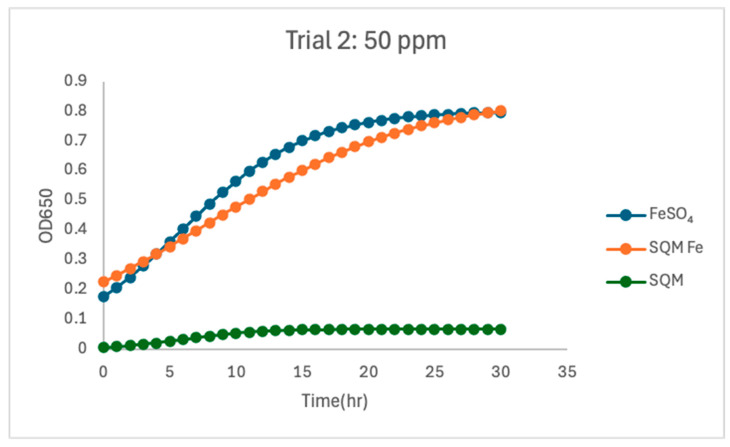
Growth dynamics for Trial 2 at 50 ppm. At 50 ppm, PIC exhibited the highest carrying capacity (K = 0.852) but a slower growth rate (µmax = 0.126), highlighting the delayed release of iron. Free iron had a slightly lower carrying capacity (K = 0.802) but a higher growth rate (µmax = 0.213), supporting substantial bacterial proliferation over time. PIC without iron again supported only minimal growth, but with a relatively high growth rate (µmax = 0.345, K = 0.069). High r^2^ values across treatments reflected predictable and consistent growth dynamics.

The current study investigated the influence of free and encapsulated iron sources on the growth parameters of *Campylobacter jejuni*. The findings provide insights into the bioavailability and utilization of iron in *Campylobacter* proliferation. The results demonstrated that free iron (FeSO_4_) consistently supported the highest growth rates (µmax) and carrying capacities (K) at higher concentrations (Figure 2, Figure 3, Figure 4, Figure 5, Figure 6 and Figure 7). These results align with previous research demonstrating the high solubility and rapid microbial uptake of FeSO_4_ in bacterial systems [13]. Iron is a critical cofactor involved in key bacterial functions, including electron transport, enzymatic activity, DNA replication, and respiration, making it indispensable for bacterial survival and growth [14].

*Campylobacter*, in particular, possesses a suite of iron acquisition systems that enable it to rapidly utilize free iron when available, including siderophore uptake pathways and iron-responsive regulators such as Fur [5,15,16]. In this study, the immediate bioavailability of FeSO_4_ enabled a rapid onset of exponential growth, particularly at 20 and 50 ppm, resulting in the highest µmax and K values observed. These results underscore the risk associated with the dietary inclusion of free iron forms in poultry production, as they may inadvertently support the robust growth of iron-dependent pathogens, such as *C. jejuni*.

### 5.2. Encapsulated Iron Restricts Immediate Growth Through Controlled Release

In contrast, encapsulated iron (PIC-Fe) exhibited a markedly different growth profile, consistent with its controlled-release design. SQM iron is manufactured using PolyTransport^®^ technology, which incorporates the mineral into a polysaccharide matrix that protects it from premature release and microbial uptake in the upper GIT, enabling targeted delivery to the small intestine for host absorption [13]. In the current study, encapsulated iron consistently demonstrated slower growth kinetics compared to FeSO_4_, with delayed increases in optical density and lower maximum specific growth rates (µmax) across all concentrations. This finding suggests that microbial access to iron was restricted by the encapsulation, reducing the capacity for rapid proliferation.

These findings align with previous observations by Hu et al. [17], who reported that controlled-release iron reduced early bacterial proliferation even when sufficient total iron was present in the system. However, it is important to note that encapsulated iron did not consistently suppress *C. jejuni* growth across all conditions and concentrations tested. At 10 ppm in Trial 2, PIC-Fe supported growth nearly equivalent to free iron, and at 50 ppm, the carrying capacity under PIC-Fe approached or exceeded that of free iron in Trial 2. These findings indicate that the growth-limiting effect of encapsulated iron is neither absolute nor uniform, and its magnitude depends on the interplay between iron concentration, release kinetics, and microbial iron demand.

### 5.3. Unique Growth Dynamics at Low Iron Levels (10 ppm)

At 10 ppm, a particularly notable and unexpected pattern emerged between trials. In Trial 1, FeSO_4_ promoted faster growth (µmax = 1.78) compared to PIC-Fe (µmax = 0.80), consistent with its rapid solubility and immediate uptake by *C. jejuni.* However, in Trial 2, encapsulated iron supported a µmax nearly identical to FeSO_4_ (µmax = 0.92). It even achieved a slightly higher carrying capacity (K = 0.11 versus 0.10). While these values may initially appear to suggest enhanced microbial support from encapsulated iron, they more accurately reflect the ability of encapsulation to gradually satisfy minimal microbial iron needs under restrictive conditions.

*C. jejuni* adapts to the environment slowly, maintaining modest growth over time. Similar dynamics have been reported in systems where regulated nutrient delivery supports metabolic activity without promoting aggressive proliferation [17]. It is important to emphasize that these findings do not suggest that encapsulated iron promotes *Campylobacter* growth; instead, they highlight the product’s functional ability to limit rapid microbial access while still delivering iron to meet basal metabolic needs over time.

Such a kinetic profile is consistent with PIC-Fe’s design objective: to avoid excess free iron in the distal gut while maintaining host bioavailability. Given that *Campylobacter* can thrive in low-iron niches and possess mechanisms to scavenge iron from complex sources [8,14], it is not surprising that delayed growth was still observed under encapsulated conditions. However, the slower onset and reduced peak densities reinforce the potential for encapsulated iron to act as a microbial growth-limiting strategy in feed formulations.

### 5.4. Trial-to-Trial Variability Demonstrates the Context-Dependent Effects of Encapsulated Iron

An important finding of this study was the variability observed between trials, particularly at 10 ppm. Under this highly restrictive iron condition, encapsulated iron did not yield fully reproducible growth responses. Rather than showing a consistent suppressive effect, the current study’s results indicate that the impact of encapsulated iron may depend on the biological context. This suggests that iron form alone may not fully determine *C. jejuni* behavior under low-iron conditions. Multiple factors may account for this inconsistency. During severe iron limitation, minor differences in iron release from the encapsulated matrix can alter the timing of exponential growth or the final biomass. Variability in inoculum physiology between trials, such as differences in pre-culture state, residual iron reserves, or stress adaptation, may have influenced the efficiency with which cells utilized encapsulated compared to free iron. Additionally, methodological differences, including chelation efficiency, media preparation, oxygen exposure, mixing, or early optical density signal behavior, may have affected the fitted growth parameters when overall growth was low.

These findings suggest the biological interpretation of encapsulated iron. The encapsulated iron did not consistently reduce *C. jejuni* growth across all conditions. Instead, its effects varied with both concentration and between trials. For instance, at 10 ppm in Trial 1, PIC-Fe exhibited a lower µmax than FeSO_4_ (1.08 versus 1.78), whereas in Trial 2, the treatments shifted that trajectory, with PIC-Fe displaying a higher µmax (0.92 versus 0.14). At 20 ppm, PIC-Fe again remained lower than FeSO_4_ in Trial 1 for both µmax and K. In Trial 2, the µmax values were nearly identical (2.25 versus 2.23), while K remained lower under PIC-Fe. Therefore, encapsulated iron altered growth kinetics relative to free iron, but not in a consistently inhibitory manner. This context dependence has practical implications for poultry nutrition and pathogen control. Rather than consistently suppressing pathogen growth, encapsulated iron may alter iron availability in ways that are less predictable for microbial utilization than soluble iron. This highlights the need to define the specific conditions under which encapsulated iron differs from free iron. Future research should directly measure iron release kinetics, more rigorously standardize the physiological state of the inoculum, and assess whether factors such as chelator strength, media composition, or microaerophilic conditions influence the response. Thus, the observed variability informs the design of subsequent experiments rather than serving solely as a limitation.

### 5.5. Implications for Poultry Production and Pathogen Control

These findings have significant implications for poultry production systems, where the form and dose of dietary iron can substantially influence the risk of pathogen colonization in the gastrointestinal tract. When dietary iron is provided in unencapsulated, soluble forms (e.g., FeSO_4_), it is rapidly absorbed in the upper GIT, but any unabsorbed iron may accumulate in the ceca and serve as a nutrient reservoir for enteric pathogens [18,19]. This risk is amplified in the presence of anti-nutritional factors, such as phytates, tannins, and insoluble fibers, which can bind dietary iron and prevent its absorption by the host—effectively increasing the pool of iron accessible to gut microbes [20,21,22]. In this context, encapsulated forms like PIC-Fe may offer dual benefits: improving host iron uptake efficiency while limiting microbial competition in the lower GIT. By shielding iron from microbial access during early digestion and delivering it directly to absorption sites, PIC-Fe may reduce the unintended consequence of iron-fueled pathogen proliferation [23]. The current study supports this concept, with encapsulated iron yielding up to 39.5% lower µmax and 31% lower K values compared to FeSO_4_ at equivalent concentrations—outcomes that reflect both the limited bioavailability of iron to *Campylobacter* and the absence of free iron excess in the environment.

### 5.6. Study Limitations and Future Directions

*Campylobacter* colonization and transmission in poultry production settings. Second, while the results suggest limited iron access in PIC-Fe treatments, the exact release profile of iron from the encapsulant under gastrointestinal conditions remains to be characterized. Future research should explore the rate and location of iron release in vivo and assess whether *C. jejuni* can upregulate siderophore systems or other transport pathways in response to prolonged iron limitation. Additionally, competitive interactions with other gut microbes, which may produce their siderophores or consume available iron, could influence the net effect of encapsulated iron on *Campylobacter* populations. Overall, the results reinforce the importance of iron sources and delivery in managing foodborne pathogens. While FeSO_4_ supports the rapid proliferation of *C. jejuni*, encapsulated iron, such as PIC-Fe, reduces microbial access to iron, thereby mitigating the risk of overgrowth. These findings contribute to a growing body of evidence supporting the use of controlled-release minerals as a strategy to balance animal nutrition with microbial control in livestock systems.

## 6. Conclusions

The current study demonstrates that free iron (FeSO_4_) consistently supported rapid and robust *Campylobacter jejuni* proliferation across all concentrations and trials. In contrast, the impact of encapsulated iron (PIC-Fe) on *C. jejuni* growth was variable and not consistently suppressive. While PIC-Fe generally yielded lower μmax and K values compared to FeSO_4_, these differences were neither uniform across iron concentrations nor reproducible between trials. Notably, at 10 ppm, PIC-Fe supported growth nearly equivalent to free iron in Trial 2, and at 50 ppm, encapsulated iron did not meaningfully limit bacterial carrying capacity. These findings indicate that encapsulated iron does not function as a reliable growth-limiting agent across all conditions tested. Rather, its effects are context-dependent and likely modulated by factors including iron concentration, matrix release kinetics, and inoculum physiology. Future work should focus on characterizing the conditions that govern PIC-Fe iron release and its interaction with *C. jejuni* iron acquisition systems before in vivo validation can meaningfully inform pathogen control strategies in poultry production.

## Figures and Tables

**Table 1:** Trial-specific µmax estimates, standard deviation, and between-trial variation for *C. jejuni* exposed to different iron sources and concentrations.

Treatment	Level	Trial	µmax	Trial Variation
FeSO_4_	10 ppm	1	1.78168592 ± 0.86326857	0.003474732
		2	0.14097197 ± 0.04114014	
SQM Fe		1	1.07775012 ± 0.63010068	0.842585435
		2	0.92379796 ± 1.36832734	
SQM		1	0.3155683 ± 0.47437121	0.275182986
		2	0.55223456 ± 0.32247705	
FeSO_4_	20 ppm	1	1.55386307 ± 0.62236604	0.123598884
		2	2.23417491 ± 0.54518847	
SQM Fe		1	1.1271794 ± 0.59446685	0.05165511
		2	2.2455566 ± 0.78828268	
SQM		1	0.55435853 ± 0.14779853	0.05662324
		2	0.24500025 ± 0.12461923	
FeSO_4_	50 ppm	1	0.21560154 ± 0.0534092	0.966576409
		2	0.21332676 ± 0.09237274	
SQM Fe		1	0.21442792 ± 0.03328387	0.009767421
		2	0.12605213 ± 0.00412055	
SQM		1	0.49119573 ± 0.12255645	0.129043435
		2	0.34507583 ± 0.12957093	

**Table 2:** Trial-specific K estimates, standard deviation, and between-trial variation for *C. jejuni* exposed to different iron sources and concentrations.

Treatment	Level	Trial	K	Trial Variation
FeSO_4_	10 ppm	1	0.07166394 ± 0.02114138	0.053779421
		2	0.10073765 ± 0.01667601	
SQM Fe		1	0.0825996 ± 0.02536771	0.179633673
		2	0.11073282 ± 0.01920469	
SQM		1	0.03200972 ± 0.04826362	0.752455076
		2	0.04026991 ± 0.01422332	
FeSO_4_	20 ppm	1	0.18091002 ± 0.06041169	0.566455038
		2	0.16209851 ± 0.03265275	
SQM Fe		1	0.12510431 ± 0.03889933	0.842960274
		2	0.12982525 ± 0.03025987	
SQM		1	0.07892371 ± 0.01307321	0.039321617
		2	0.03614538 ± 0.01942359	
FeSO_4_	50 ppm	1	0.64159871 ± 0.08182207	0.966576409
		2	0.80265801 ± 0.1129353	
SQM Fe		1	0.49851146 ± 0.05981793	3.94 × 10^6^
		2	0.85248181 ± 0.01586402	
SQM		1	0.08707481 ± 0.02065056	0.252939205
		2	0.06945285 ± 0.02140918	

**Table 3:** Trial-specific R^2^ estimates, standard deviation, and between-trial variation for *C. jejuni* exposed to different iron sources and concentrations.

Treatment	Level	Trial	R^2^	Trial Variation
FeSO_4_	10 ppm	1	0.6454911 ± 0.37857334	0.083259855
		2	0.98121524 ± 0.00668497	
SQM Fe		1	0.9448633 ± 0.01985567	0.289531745
		2	0.92096032 ± 0.03737288	
SQM		1	0.30410664 ± 0.42995563	0.869565688
		2	0.21737945 ± 0.80023302	
FeSO_4_	20 ppm	1	0.89734231 ± 0.09578924	0.718420676
		2	0.85860974 ± 0.1857391	
SQM Fe		1	0.91372267 ± 0.08019936	0.916187674
		2	0.91928484 ± 0.07269594	
SQM		1	0.96373193 ± 0.03070705	0.058597461
		2	0.67826477 ± 0.11112874	
FeSO_4_	50 ppm	1	0.95262067 ± 0.03676922	0.446321121
		2	0.96773749 ± 0.01874198	
SQM Fe		1	0.93464114 ± 0.04281943	0.118425856
		2	0.96820999 ± 0.0030273	
SQM		1	0.97653632 ± 0.00898097	0.153208155
		2	0.87142637 ± 0.12916084	

## Data Availability

The original contributions presented in this study are included in the article. Further inquiries can be directed to the corresponding author.
